# Receipt of First and Second Doses of JYNNEOS Vaccine for Prevention of Monkeypox — United States, May 22–October 10, 2022

**DOI:** 10.15585/mmwr.mm7143e2

**Published:** 2022-10-28

**Authors:** Jennifer L. Kriss, Peter M. Boersma, Emalee Martin, Kirsten Reed, Jennifer Adjemian, Nathaniel Smith, Rosalind J. Carter, Kathrine R. Tan, Arunkumar Srinivasan, Sunanda McGarvey, Jennifer McGehee, Danielle Henderson, Noah Aleshire, Adi V. Gundlapalli

**Affiliations:** ^1^CDC Monkeypox Emergency Response Team; ^2^Office of Informatics, National Center for Immunization and Respiratory Diseases, CDC; ^3^Peraton, Inc., Herndon, Virginia.

Vaccination with JYNNEOS vaccine (Modified Vaccinia Ankara vaccine, Bavarian Nordic) to prevent monkeypox commenced shortly after confirmation of the first monkeypox case in the current outbreak in the United States on May 17, 2022 (*1*). To date, more than 27,000 cases have been reported across all 50 states, the District of Columbia (DC), and Puerto Rico.* JYNNEOS vaccine is licensed by the Food and Drug Administration (FDA) as a 0.5-mL 2-dose series administered subcutaneously 28 days apart to prevent smallpox and monkeypox infections (*2*) and has been found to provide protection against monkeypox infection during the current outbreak (*3*). The U.S. Department of Health and Human Services (HHS) allocated 1.1 million vials of JYNNEOS vaccine from the Strategic National Stockpile, with doses allocated to jurisdictions based on case counts and estimated size of population at risk (*4*). However, initial vaccine supplies were severely constrained relative to vaccine demand during the expanding outbreak. Some jurisdictions with highest incidence responded by prioritizing first dose administration during May–July (*5*,*6*). The FDA emergency use authorization (EUA) of 0.1 mL dosing for intradermal administration of JYNNEOS for persons aged ≥18 years on August 9, 2022, substantially expanded available vaccine supply^†^ (*7*). The U.S. vaccination strategy focuses primarily on persons with known or presumed exposures to monkeypox (*8*) or those at high risk for occupational exposure (*9*). Data on monkeypox vaccine doses administered and reported to CDC by U.S. jurisdictions were analyzed to assess vaccine administration and completion of the 2-dose series. A total of 931,155 doses of JYNNEOS vaccine were administered and reported to the CDC by 55 U.S. jurisdictions during May 22–October 10, 2022. Among persons who received ≥1 dose, 51.4% were non-Hispanic White (White), 22.5% were Hispanic or Latino (Hispanic), and 12.6% were non-Hispanic Black or African American (Black). The percentages of vaccine recipients who were Black (5.6%) and Hispanic (15.5%) during May 22–June 25 increased to 13.3% and 22.7%, respectively, during July 31–October 10. Among 496,888 persons who received a first dose and were eligible for a second dose during the study period, 57.6% received their second dose. Second dose receipt was highest among older adults, White persons, and those residing in the South U.S. Census Bureau Region. Tracking and addressing disparities in vaccination can reduce inequities, and equitable access to and acceptance of vaccine should be an essential factor in planning vaccination programs, events, and strategies. Receipt of both first and second doses is necessary for optimal protection against *Monkeypox virus* infection.

The system for reporting monkeypox vaccination data was adapted from existing infrastructure and data flow structures developed for reporting COVID-19 vaccine administration data.^§^ Providers submitted monkeypox vaccination data to their jurisdiction’s immunization information systems (IIS); state, local, and territorial jurisdictions reported vaccine administration data to CDC. Monkeypox vaccine doses administered to persons in 49 states, New York City, Philadelphia, DC, Puerto Rico, the U.S. Virgin Islands, and the Northern Mariana Islands during May 22–October 10, 2022, and reported to CDC as of October 12, 2022, were analyzed to assess vaccination by sex, age group, race and ethnicity, U.S. Census Bureau region, and urbanicity.^¶^

The proportion of persons who received a second JYNNEOS vaccine dose was calculated from among all persons who received a first dose and were due for their second dose during the study period.** First and second doses were linked by CDC based on a recipient identifier assigned by the reporting entity and the three-digit reporting entity code.^††^ The interval in days between first and second doses was calculated using date of administration for each dose. Analyses were conducted using SQL Server Management Studio (version 18; Microsoft) and SAS software (version 9.4; SAS Institute). This activity was reviewed by CDC and was conducted consistent with applicable federal law and CDC policy.^§§^

During May 22–October 10, 2022, a total of 931,155 JYNNEOS vaccine doses were administered and reported to CDC by U.S. jurisdictions, including 628,610 (67.5%) first doses, 301,770 (32.4%) second doses, and 775 (0.1%) third, fourth, or fifth doses.^¶¶^ Weekly first dose administration peaked at 102,262 during the week August 7–13 (Supplementary Figure, https://stacks.cdc.gov/view/cdc/121818). The majority of vaccine doses (63%) were administered in the six states reporting the highest monkeypox case counts (California, Florida, Georgia, Illinois, New York, and Texas).***

Among 628,610 persons who received ≥1 dose of vaccine, 91.9% were male, and 65.4% were aged 25–49 years (Table 1). Most vaccine recipients were residents of urban counties (82.5%); 15.9% and 1.6% lived in suburban and rural counties, respectively. Race and ethnicity were reported for 91.0% of vaccinated persons; among those, 51.4% were White, 22.5% were Hispanic, 12.6% were Black, and 7.6% were Asian persons. The percentages of vaccine recipients who were Black (5.6%) and Hispanic (15.5%) during May 22–June 25, increased to 9.4% and 21.9%, respectively, during June 26–July 30, and to 13.3% and 22.7%, respectively, during July 31–October 10 (Figure) (Supplementary Table 1, https://stacks.cdc.gov/view/cdc/121816). The most common provider sites where persons received vaccines were public health clinics (41.5%), commercial vaccination service providers (13.6%), medical practices (9.3%), and hospitals (9.1%) (Table 1).

**TABLE 1:** Characteristics of persons who have received first and second doses of JYNNEOS vaccine — United States, May 22-October 10, 2022

Characteristic	No. (%)*	
	First dose^†^	Second dose
**Total**	**628,610 (100)**	**301,770 (100)**
**Sex**		
Male	567,457 (91.9)	282,486 (94.8)
Female	49,944 (8.1)	15,405 (5.2)
Unknown	11,209 (—)	3,879 (—)
**Age group, yrs**		
0–4	218 (0.03)	39 (0.01)
5–11	315 (0.1)	55 (0.02)
12–17	418 (0.1)	80 (0.03)
18–24	48,824 (7.8)	16,358 (5.4)
25–39	296,931 (47.2)	137,787 (45.7)
40–49	114,337 (18.2)	59,021 (19.6)
50–64	132,942 (21.1)	70,496 (23.4)
≥65	34,619 (5.5)	17,934 (5.9)
Unknown	6 (—)	0 (—)
**Race and ethnicity** ^§^		
AI/AN, non-Hispanic	2,194 (0.4)	882 (0.3)
Asian, non-Hispanic	43,266 (7.6)	20,308 (7.2)
Black or African American, non-Hispanic	71,855 (12.6)	33,948 (12.1)
Hispanic or Latino	128,853 (22.5)	58,288 (20.8)
NH/OPI, non-Hispanic	1,486 (0.3)	659 (0.2)
White, non-Hispanic	293,853 (51.4)	152,435 (54.3)
Multiracial/Other, non-Hispanic	30,643 (5.4)	14,298 (5.1)
Unknown	56,460 (—)	20,952 (—)
**U.S. Census Bureau region** ^¶^		
Northeast	164,357 (26.3)	74,526 (24.8)
Midwest	70,419 (11.3)	33,640 (11.2)
South	173,783 (27.8)	98.695 (32.9)
West	217,276 (34.7)	93,503 (31.1)
Urbanicity**		
Urban	482,865 (82.5)	228,685 (80.5)
Suburban	93,113 (15.9)	51,041 (18.0)
Rural	9,418 (1.6)	4,297 (1.5)
Unknown	40,439 (—)	16,341 (—)
**Location type of vaccine administration**		
Public health provider, public health clinic	225,040 (41.5)	103,818 (39.7)
Commercial vaccination service provider	73,783 (13.6)	47,835 (18.3)
Medical practice	50,659 (9.3)	22,504 (8.6)
Hospital	49,400 (9.1)	16,767 (6.4)
Public health provider, FQHC	34,118 (6.3)	17,049 (6.5)
Health center, community	17,311 (3.2)	9,470 (3.6)
Health center, other	16,835 (3.1)	7,439 (2.8)
Pharmacy	16,834 (3.1)	9,039 (3.5)
Other	58,065 (10.7)	27,279 (10.4)
Unknown	86,565 (—)	40,570 (—)

**Abbreviations**: AI/AN = American Indian or Alaska Native; FQHC = Federally Qualified Health Center; NH/OPI = Native Hawaiian or other Pacific Islander.

* Percentages calculated using nonmissing data.

^†^ Overall, 775 doses were reported as third, fourth, or fifth doses.

^§^ Persons with Hispanic or Latino (Hispanic) ethnicity were categorized as Hispanic and might be of any race; persons with non-Hispanic ethnicity were categorized as non-Hispanic American Indian or Alaska Native, Asian, Black or African American, Native Hawaiian or other Pacific Islander, White, multiracial (more than one race category selected), or other. Persons with missing data for either race or ethnicity were categorized as unknown race and ethnicity.

^¶^ For each U.S. Census Bureau region, vaccine allocations (vials) were 251,150 (Northeast); 404,026 (South); 148,419 (Midwest); and 281,975 (West) (https://aspr.hhs.gov/SNS/Pages/JYNNEOS-Distribution.aspx). A total of 2,775 vaccine recipients were in U.S. territories and freely associated states and were not categorized in a U.S. Census Bureau region.

**Urbanicity was classified based on the vaccine recipient’s county of residence using the National Center of Health Statistics’ 2013 Urban-Rural Classification Scheme for Counties: urban includes large central metropolitan, medium metropolitan, and small metropolitan counties; suburban includes large fringe metropolitan counties; and rural includes micropolitan and noncore counties (https://www.cdc.gov/nchs/data_access/urban_rural.htm). A total of 40,439 vaccine recipients had an unknown or missing county of residence; 2,775 vaccine recipients were in U.S. territories and freely associated states and were not categorized as urban, suburban, or rural.

**FIGURE:**
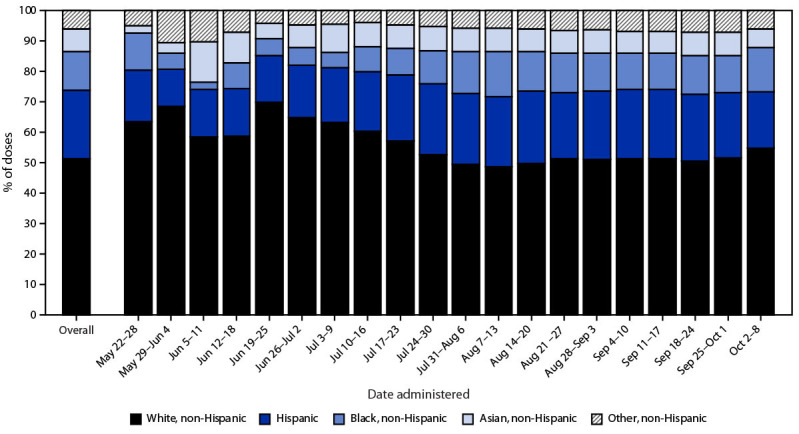
Race and ethnicity*^,^^†^ of persons who received ≥1 dose of JYNNEOS vaccine, by week of administration — United States, May 22–October 8, 2022 * Race and ethnicity were missing for 56,460 (9.0%) vaccinated persons. ^†^ Persons with Hispanic or Latino (Hispanic) ethnicity were categorized as Hispanic and might be of any race; persons with non-Hispanic ethnicity were categorized as non-Hispanic American Indian or Alaska Native, Asian, Black or African American, Native Hawaiian or other Pacific Islander, White, multiracial (more than one race category selected), or other. Persons with missing data for either race or ethnicity were categorized as unknown race and ethnicity.

Among 496,888 first-dose vaccine recipients who were eligible to receive a second dose during the study period, 285,964 (57.6%) had received the second dose as of October 10 (Table 2). Receipt of a second dose was highest in the South (70.0%) and lowest in the Northeast (51.8%). The percentage of persons who received a second dose varied across jurisdictions (range = 22.4%–82.5%) (Supplementary Table 2, https://stacks.cdc.gov/view/cdc/121817). In New York City and Philadelphia, where administration of second doses was delayed because of prioritization of first doses (*5*,*6*), fewer than one half of first-dose recipients had received a second dose. Second dose receipt was lower among females (39.4%) than among males (59.3%), was highest among White persons (61.4%), was lowest among persons of Hispanic (53.9%) or unknown race or ethnicity (51.2%), and increased with increasing age. More than one half of eligible Black, Asian, American Indian or Alaska Native, and Native Hawaiian or other Pacific Islander persons received a second dose. Among persons who received a second dose, 68.7% received the dose within the recommended interval of 24–35 days after the first dose (median = 31 days; IQR = 28–38 days) (Table 2).

**TABLE 2:** Receipt of second dose of JYNNEOS vaccine among persons who initiated the JYNNEOS vaccination series — United States, May 22–October 10, 2022

Characteristic	No. (%) who received a second dose*^,†^	Median interval between first and second dose (days)^§^	% of second doses administered within recommended interval^¶^
Total**	**285,964 (57.6)**	**31**	**68.7**
**Sex**			
Male	267,508 (59.3)	31	68.3
Female	14,382 (39.4)	29	76.1
Unknown	4,074 (45.3)	30	68.6
**Age group, yrs**			
0–4	38 (26.0)	28	84.2
5–11	52 (25.2)	29	90.4
12–17	75 (31.9)	28	88.0
18–24	15,486 (41.2)	31	68.2
25–39	130,618 (54.2)	32	64.5
40–49	55,805 (61.6)	31	69.3
50–64	66,849 (65.6)	29	73.9
≥65	17,041 (67.1)	29	78.5
**Race and ethnicity^††^**			
AI/AN, non-Hispanic	883 (55.9)	30	71.8
Asian, non-Hispanic	19,559 (55.5)	33	60.2
Black or African American, non-Hispanic	31,590 (55.4)	30	72.0
Hispanic or Latino	54,483 (53.9)	31	68.0
NH/OPI, non-Hispanic	653 (54.1)	31	69.1
White, non-Hispanic	144,112 (61.4)	31	68.8
Multiracial/Other, non-Hispanic	11,611 (55.8)	31	68.9
Unknown	23,073 (51.2)	31	71.6
**U.S. Census Bureau region^§§^**			
Northeast	73,324 (51.8)	37	47.1
Midwest	32,433 (59.3)	29	77.1
South	85,859 (70.0)	28	83.8
West	93,003 (52.9)	31	68.5

**Abbreviations**: AI/AN = American Indian or Alaska Native; NH/OPI = Native Hawaiian or other Pacific Islander.

* Among persons who received a first dose ≥28 days earlier (496,888).

^†^ Analysis of second dose receipt incorporates 7-day lag to account for reporting delays between vaccine administration and data report to CDC.

^§^ Among persons who received a second dose.

^¶^ 24–35 days after the first dose.

** Texas did not submit person-level vaccination data; therefore, persons who received ≥1 dose in Texas (23,264) were excluded from analysis of second dose receipt.

^††^ Persons with Hispanic or Latino (Hispanic) ethnicity were categorized as Hispanic and might be of any race; persons with non-Hispanic ethnicity were categorized as non-Hispanic American Indian or Alaska Native, Asian, Black or African American, Native Hawaiian or other Pacific Islander, White, multiracial (more than one race category selected), or other. Persons with missing data for either race or ethnicity were categorized as unknown race and ethnicity.

^§§^ A total of 1,345 persons were in U.S. territories and freely associated states and were not categorized in a U.S. Census Bureau region.

## Discussion

This report documents the first large-scale effort to provide JYNNEOS vaccine to persons at higher risk for exposure to *Monkeypox virus* in the United States. More than 900,000 doses of JYNNEOS vaccine were administered during the first 5 months of the vaccination effort, with approximately 628,000 persons receiving ≥1 dose, and 302,000 persons receiving the complete 2-dose series. Although a peak in first dose administration occurred in mid-August, administration of first and second vaccine doses is continuing. More than one half of persons who initiated the vaccination series and were eligible for a second dose during the study period have received their second dose; completion of the second dose is necessary for optimal protection against *Monkeypox virus* infection. Importantly, there was substantial progress in increasing the proportion of Black and Hispanic persons vaccinated during the more recent period. Increasing the availability of vaccine at community events, including a focus on health equity, has contributed to these improvements.

The findings in this report are subject to at least four limitations. First, it was not possible to assess vaccination based on gender identity because this information is not routinely collected during vaccine administration, and existing IIS systems do not include this variable. Second, race or ethnicity was unknown for 9.0% of persons who received JYNNEOS vaccine, which could limit ability to interpret differences by race and ethnicity. Third, linkage of an individual person’s first and second doses depends on the accuracy of recipient identifiers in a jurisdiction’s IIS. Persons who received their second dose in a different jurisdiction than where they received their first dose might not be able to be linked to their first dose, resulting in an underestimation of second dose receipt. Finally, second-dose status was unknown for 23,264 (3.7%) first-dose recipients who lived in a jurisdiction that did not submit person-level vaccination data; vaccine recipients from this jurisdiction were not included in the analysis of second dose receipt.

HHS allocated and distributed vaccine doses to prioritize persons at highest risk for exposure to *Monkeypox virus* and in jurisdictions with the highest case counts and size of priority population. The multiphase allocation and distribution of vaccines was necessary because of initial supply limitations during the period of most rapid epidemic growth. Jurisdictions developed vaccination strategies based on local epidemiology and availability of resources. Early constraints might have limited vaccine access for populations who could not travel long distances, had inflexible work schedules, or could not access online appointment scheduling. Over time, jurisdictions have worked to improve vaccine access, including increasing the number of vaccine events and providers. Many persons with increased risk factors during the current monkeypox outbreak have received ≥1 dose of monkeypox vaccine. However, analysis of the demographic characteristics of persons who have received vaccine indicates that there are certain demographic groups who were less likely to be vaccinated, including Hispanic and Black persons, despite being disproportionately affected by the monkeypox outbreak (*10*). Monitoring and addressing disparities in vaccine administration can reduce health inequities. CDC’s monkeypox vaccine equity pilot program provides an opportunity to implement and evaluate novel strategies to reach populations most affected by the monkeypox outbreak but who might face barriers to getting vaccinated.^†††^ Equitable access to and acceptance of vaccine should be an essential factor in planning vaccination programs, events, and strategies. Receipt of both first and second doses is necessary for optimal protection against *Monkeypox virus* infection. Improving equity in vaccination for both first and second doses is important to protect persons who are most at risk and to end the current monkeypox outbreak.

**Summary**

**What is already known about this topic?**

In the United States, JYNNEOS vaccine is recommended for persons exposed to or at high risk for exposure to *Monkeypox virus*.

**What is added by this report?**

By October 10, 2022, a total of 931,155 JYNNEOS vaccine doses were administered in the United States. Among persons who received ≥1 vaccine dose, 51.4% were non-Hispanic White, 12.6% were non-Hispanic Black or African American (Black), and 22.5% were Hispanic persons. The percentages of vaccine recipients who were Black (5.6%) and Hispanic (15.5%) during May 22–June 25 increased to 13.3% and 22.7%, respectively, during July 31–October 10.

**What are the implications for public health practice?**

Tracking and addressing disparities in vaccination can reduce inequities and help ensure that disproportionately affected populations are protected.

## Footnotes

*
https://www.cdc.gov/poxvirus/monkeypox/response/2022/us-map.html

† During May–September 2022, 833,293 vials of JYNNEOS vaccine were shipped to U.S. jurisdictions. Before the EUA for intradermal administration of JYNNEOS on August 9, 2022, one vial contained 1 dose of vaccine; under the EUA, when 0.1 mL dosing and intradermal administration are used, one vial contains up to 5 doses of vaccine. The U.S. jurisdictions that received vaccine were the 50 states, DC, five local jurisdictions that received vaccine separately from their respective states (Los Angeles, California; Chicago, Illinois; New York, New York; Philadelphia, Pennsylvania; and Houston, Texas), and eight U.S. territories or freely associated states (American Samoa, Federated States of Micronesia, Guam, Marshall Islands, Northern Mariana Islands, Palau, Puerto Rico, and the U.S. Virgin Islands).

§
https://www.cdc.gov/coronavirus/2019-ncov/vaccines/distributing/about-vaccine-data.html

¶ Urbanicity was classified based on the vaccine recipient’s county of residence using the National Center for Health Statistics’ 2013 Urban-Rural Classification Scheme for Counties: urban includes large central metropolitan, medium metropolitan, and small metropolitan counties; suburban includes large fringe metropolitan counties; and rural includes micropolitan and noncore counties. https://www.cdc.gov/nchs/data_access/urban_rural.htm

** Persons were considered to be due for their second dose if they received their first dose ≥28 days earlier. Analysis of second dose receipt incorporates a 7-day lag to account for reporting delays between vaccine administration and data report to CDC.

†† Texas did not submit person-level vaccination data; therefore, the 23,264 persons who received ≥1 dose in Texas were excluded from the analysis of second dose receipt.

§§ 45 C.F.R. part 46, 21 C.F.R. part 56; 42 U.S.C. Sect. 241(d); 5 U.S.C. Sect. 552a; 44 U.S.C. Sect. 3501 et seq.

¶¶ Third, fourth, or fifth doses might have been administered if an administration error occurred and a dose had to be repeated; alternatively, if the same recipient identifier was assigned to multiple persons in a jurisdiction, doses might have been erroneously considered to be third, fourth, or fifth doses. https://www.cdc.gov/poxvirus/monkeypox/interim-considerations/errors-deviations.html

***
https://www.cdc.gov/poxvirus/monkeypox/response/2022/us-map.html

†††
https://www.cdc.gov/poxvirus/monkeypox/health-departments/vaccine-equity-pilot.html
